# The Critical Role of Inflammation in the Pathogenesis and Progression of Myeloid Malignancies

**DOI:** 10.3390/cancers10040104

**Published:** 2018-04-03

**Authors:** Brianna M. Craver, Kenza El Alaoui, Robyn M. Scherber, Angela G. Fleischman

**Affiliations:** 1 Department of Biological Chemistry, University of California Irvine, Irvine, CA 92697, USA; bcraver@uci.edu; 2 Department of Internal Medicine, Université Libre de Bruxelles, 1050 Brussels, Belgium; kenza.el.alaoui@ulb.ac.be; 3 Department of Medicine, University of California Irvine, Irvine, CA 92697, USA; 4 Department of Hematology and Oncology, Mays MD Anderson Cancer Center, University of Texas San Antonio, San Antonio, TX 78249, USA; scherber@uthscsa.edu

**Keywords:** myeloid malignancy, clonal hematopoiesis, hematopoietic stem cells, symptom burden, inflammation

## Abstract

Hematopoietic stem cells (HSCs) maintain an organism’s immune system for a lifetime, and derangements in HSC proliferation and differentiation result in hematologic malignancies. Chronic inflammation plays a contributory if not causal role in HSC dysfunction. Inflammation induces HSC exhaustion, which promotes the emergence of mutant clones that may be resistant to an inflammatory microenvironment; this likely promotes the onset of a myeloid hematologic malignancy. Inflammatory cytokines are characteristically high in patients with myeloid malignancies and are linked to disease initiation, symptom burden, disease progression, and worsened prognostic survival. This review will cover our current understanding of the role of inflammation in the initiation, progression, and complications of myeloid hematologic malignancies, drawing from clinical studies as well as murine models. We will also highlight inflammation as a therapeutic target in hematologic malignancies.

## 1. Introduction

Inflammation drives tumor progression in multiple cancer types [1]. While the role of inflammation in the initiation and progression of many solid tumors is well established, our understanding of how inflammation promotes initiation and progression of hematologic malignancies is an emerging field. Hematologic malignancies arise from the expansion of mutated hematopoietic stem cell (HSC) clones with uncontrolled proliferation or differentiation. Myeloid hematologic malignancies can manifest with a wide range of phenotypes. Acute myeloid leukemia (AML) is characterized by a blockade in differentiation with explosive expansion of immature hematopoietic cells (blasts) that is rapidly fatal without treatment. In myeloid diseases with a more chronic course, such as myeloproliferative neoplasms (MPN) and chronic myelogenous leukemia (CML), differentiation is intact but there is aberrant expansion of mature myeloid cells. In myelodysplastic syndrome (MDS), hematopoietic precursors are expanded in the bone marrow, but there is ineffective hematopoiesis leading to low peripheral blood counts. In order for a myeloid malignancy to arise, the mutated HSC must outcompete the non-mutated competitor HSCs. Therefore, the selective pressures imposed upon an organism’s HSC pool define which mutant clones will have a selective advantage and expand at the expense of their normal counterparts. Chronic inflammation is a common selective pressure that leads to impaired fitness of normal HSCs. In this review, we will highlight how inflammation drives the emergence of mutant HSC clones, promotes myeloid malignancy progression, and exacerbates symptom burden. A clear understanding of how inflammation drives each of these processes is key to developing interventions that prevent disease initiation, halt progression, and improve quality of life in myeloid malignancies.

## 2. Inflammation Is a Clinical Concern for Myeloid Malignancies

### 2.1. Chronic Inflammation Is a Shared Characteristic in Myeloid Malignancies

**Figure 1:**
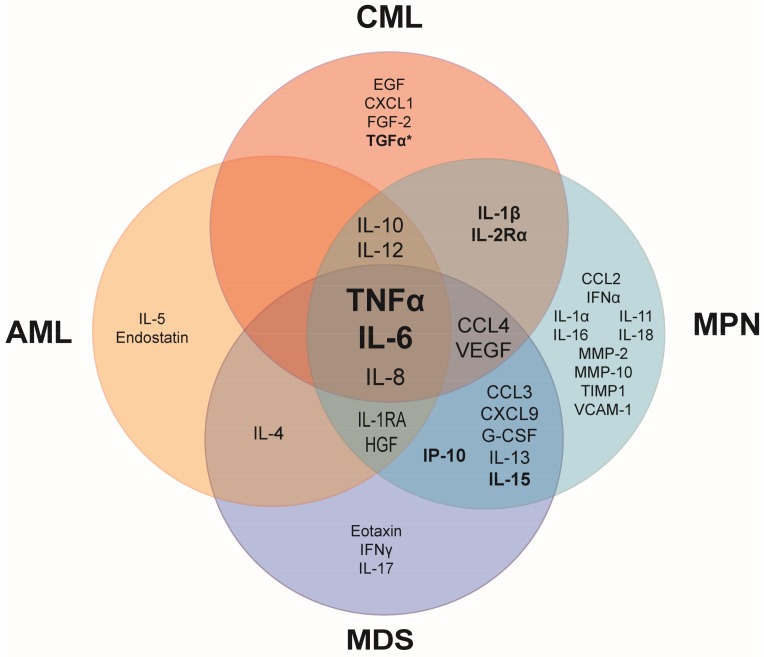
Common upregulated pro- and anti-inflammatory cytokines in patient blood plasma or serum compared to healthy controls. Elevated tumor necrosis factor alpha (TNFα) and interleukin 6 (IL-6) are shared among the major myeloid malignancies. Cytokines in bold have been linked to prognostic survival in at least one hematopoietic malignancy. *Prognostic survival is correlated with transforming growth factor alpha (TGFα) only when IL-6 is also elevated. AML, acute myeloid leukemia; CML, chronic myeloid leukemia; MDS, myelodysplastic syndrome; MPN, myeloproliferative neoplasm. Referenced in [2,3,4,5,6,7,8,9]. EGF: epidermal growth factor; CXCL: chemokine (C-X-C motif) ligand; FGF: fibroblast growth factor; IL-1RA: interleukin 1 receptor antagonist; HGF: hepatocyte growth factor; IFNα: interferon alpha; CCL: chemokine (C-C motif) ligand; VEGF: vascular endothelial growth factor; G-CSF: granulocyte colony-stimulating factor; MMP: matrix metalloproteinase; TIMP: tissue inhibitor of metalloproteinases; VCAM: vascular cell adhesion molecule.

A shared characteristic of many hematologic malignancies is the overproduction of inflammatory cytokines. Although several cytokines are overexpressed in myeloid malignancies, overproduction of tumor necrosis factor alpha (TNFα) and interleukin 6 (IL-6) is most commonly observed in patients, suggesting that these cytokines play a role in the fundamental aspects of the development and/or manifestations of hematologic malignancies (Figure 1). Some cytokines are uniquely elevated in specific myeloid malignancies, suggesting that these cytokines may have a specific role in the pathogenesis of the hematologic malignancy in which they are overexpressed. In myeloid malignancies, the anti-inflammatory cytokines, IL-4, IL-10 and IL-13 are also commonly elevated in patients (Figure 1), likely as a response to the chronic inflammatory state in an attempt to dampen inflammation. 

Elevated cytokines are commonly linked to a worsened prognosis, particularly in MPN, as indicated by the bolded biomarkers in Figure 1. In treatment-naive MPN patients, increased levels of IL-8, IL-2R, IL-12, IL-15, and IP-10 were independently predictive of inferior survival [2]. A normal age-related phenomenon is that inflammatory cytokines increase with age (referred to as inflamm-aging). Aging-induced inflammation likely plays a role in initiation as well as progression of myeloid malignancies. One study compared plasma cytokine levels in AML patients that were younger or older than 65 years of age versus aged-matched normal controls. In agreement with previous literature, IL-6 and IL-8 were significantly elevated in older healthy patients compared to younger healthy patients [3]. However, AML patients with elevated IL-6 and IL-17A had an even worse prognostic survival compared to AML patients with only high IL-6. This finding suggests that cytokines play synergistic roles, which may in part explain why prognostic survival is correlated with multiple elevated cytokines rather than just a single elevated cytokine in AML and other malignancies [3]. Further, AML patients with elevated IL-10 and decreased IL-6 were correlated with improved patient survival, demonstrating the importance of maintaining a balance between pro- and anti-inflammatory cytokines [3]. 

Inflammatory cytokines can be used as a predictor of patient response to therapies. Nievergall and colleagues reported that newly diagnosed CML patients exhibited several elevated inflammatory cytokines in plasma compared to healthy controls [4] (Figure 1). Following three months of treatment with imatinib, a BCR-ABL kinase inhibitor, cytokine profiles from patients that were early molecular responders (EMR) were compared to patients that failed to achieve EMR. Strikingly, transforming growth factor alpha (TGFα) was uniquely elevated in the CML patients that failed EMR and was a more significant predictor of failed response to imatinib treatment than IL-6. While TGFα has long been investigated and linked to prognostic survival in many solid tumor types, this is the first evidence that TGFα is an important cytokine in a hematologic malignancy [4].

### 2.2. Autoinflammatory and Autoimmune Diseases in Hematological Malignancies

Many studies have demonstrated an association between cancer and autoimmune diseases (AID). Elevated pro-inflammatory cytokines are found in the serum of patients diagnosed with AID. These cytokines likely play a critical role in the pathogenesis of AID and may also contribute to autoimmune-associated tumorigenesis [10]. Although the majority of studies on cancer and autoimmunity focus on solid tumors, some studies have highlighted a specific association with hematologic malignancies. For example, specific autoimmune disorders such as systemic lupus erythematosus or autoimmune hemolytic anemia display a major risk of Non-Hodgkin Lymphoma [11]. Moreover, a chronic immune stimulation has been considered as a possible trigger for hematologic malignancies such as MDS and AML, which were increased significantly in patients with a broad range of autoimmune conditions [12,13]. Specifically, AML was most closely associated with systemic lupus erythematosus, rheumatoid arthritis, polymyalgia rheumatica, and autoimmune hemolytic anemia while MDS was associated with pernicious anemia and rheumatoid arthritis [12]. 

There is a growing body of evidence linking a genetic predisposition to chronic inflammation with MPN [14]. MPN patients and their unaffected family members have an increased incidence of autoimmune diseases [15,16]. Genome-wide association studies (GWAS) of MPN [17,18,19] and inflammatory diseases have identified associations with the same genes. For example, the Janus kinase 2 (JAK2) single nucleotide polymorphism (SNP) rs10758669, a SNP that tags the 46/1 haplotype associated with JAK2^V617F^-mutated MPN, was also identified previously to be associated with Crohn’s disease [20]. It has been proposed that the JAK2 46/1 haplotype results in an augmented response to cytokine stimulation, leading to increased inflammation [21]. Interestingly, a study found JAK2^V617F^ in about 20% of people with mild thrombocytosis in an inflammatory bowel disease (IBD) clinic, suggesting that a significant fraction of people with IBD harbor a JAK2^V617F^ clone [22]. In addition, MPN patients have a >2-fold increased risk of IBD, a result that links these two disease entities with potentially common predispositions. Moreover, SNPs in SH2B3 (Lnk) are associated with MPN [17], multiple sclerosis and atherosclerosis [23], though atherosclerosis is not an autoimmune disease but a chronic inflammatory state.

Autoinflammatory diseases are characterized by recurrent inflammatory episodes without any evidence of auto-antibodies, making them distinct from the autoimmune conditions. These diseases include Familial Mediterranean Fever (FMF), periodic fever, aphthous stomatitis, pharyngitis, and adenitis syndrome (PFAPA), or adult Still’s disease [24]. As these clinical entities are rare and less studied than AID, very few studies have investigated the incidence of cancer in these populations with a recurring inflammatory environment. Interestingly, a recent study on Israeli FMF patients showed a decreased cancer incidence compared to the general population [25]. Given that FMF is a chronic inflammatory state, one would expect that patients with FMF would have higher rather than lower cancer incidence. Further studies must be performed using a larger population of FMF patients to resolve these uncertainties. If FMF patients truly have a decreased incidence of cancer compared to the populous then it may be worth identifying whether particular circulating cytokines are responsible for this protective effect.

The relationship between hematologic malignancies and AID is bidirectional, as lymphoid and myeloid neoplasms classically present autoimmune manifestations, sometimes even revealing them. AID can also be triggered by treatments for hematologic malignancies; many chemotherapies and immune agents have been implicated in the development of autoimmune cytopenias [26]. Some hematological diseases can also be uniquely driven by an autoimmune pathogenesis. Primary autoimmune myelofibrosis has a benign course and is distinct from a neoplastic process, as seen in primary myelofibrosis or secondary to chronic myeloproliferative neoplasm [27]. The key difference between autoimmune myelofibrosis and MPN myelofibrosis is that a mutated HSC clone drives MPN myelofibrosis whereas in autoimmune myelofibrosis there is no evidence of a neoplastic clone.

### 2.3. Inflammation Exacerbates Symptom Burden in Myeloid Malignancies

Chronic inflammation worsens cancer-related symptoms [28]. Elevated proinflammatory cytokines such as TNFα, IL-6 and IL-1 are correlated with sickness behavior which consists of lethargy, depression, anorexia, anhedonia, cognitive impairment, hyperalgesia, and decreased social interaction [29]. This behavior has developed as an adaptive reaction during times of acute infection, but when these cytokines are chronically elevated in the context of cancer it has a significantly negative impact on quality of life. The impact of chronic inflammation on disease burden is particularly obvious in chronic MPNs which include polycythemia vera (PV), essential thrombocythemia (ET), and primary myelofibrosis (PMF). MPNs most commonly result from somatic mutations in HSCs in JAK2, calreticulin, or MPL, with the central theme being constitutive activation of thrombopoietin receptor (MPL) and downstream activation of the Janus kinases and Signal Transducer and Activator of Transcription proteins (JAK/STAT) signaling pathway. In MPN patients, a plethora of inflammatory cytokines is elevated (demonstrated in Figure 1) and drives many of the debilitating symptoms associated with the disease [30,31]. 

The impact that physical symptoms have on quality of life and general well being in patients with hematologic malignancies is under-recognized, as most therapies are focused on blood counts rather than symptoms. Recent studies have revealed how much the physical symptom burden weighs on the psychology of patients with hematologic disorders [32], and how this diagnosis can lead to distress, depression, and anxiety [33]*.* In aggressive hematologic malignancies such as AML, the major focus is on eradication of the leukemic clone because the consequence of not targeting the mutant clone is fatal. On the other hand, for chronic hematologic malignancies such as MPN, only a few MPN patients will inevitably fail treatments and see their disease progress rapidly or transform into acute leukemia. Because MPN is a chronic disease, the majority of MPN patients live with this diagnosis with ‘watch and wait’ management. However, this approach is being challenged by the renaissance of interferon-alpha (IFNα) [34,35]. IFNα depletes MPN disease-initiating cells by inducing cell cycle activation of mutant HSCs and driving them to erythroid-lineage differentiation [36]. IFNα has been used for decades, even in low-risk patients, and can lead to molecular responses in a both JAK2 and CALR mutated MPN patients; moreover, these responses can be maintained for several years after discontinuation of IFNα [37,38,39,40]. In addition, IFNα therapy can blunt or even reverse fibrosis in early stage MF patients [41]. The ability to modify disease course is unique to IFNα, in particular, at the early stages of disease, however its side effect profile poses a challenge to more widespread use. Ropeginterferon, a long-acting IFNα with a tolerable toxicity profile, is currently being evaluated in clinical trials [42]. The availability of an easily tolerated IFNα therapy may lead to more widespread use of IFNα in early disease MPN patients. Regardless, given that symptom burden has a major impact on the quality of life of MPN patients, there should be a focus on identifying therapeutics for managing symptoms in addition to targeting malignant HSC clones.

**Figure 2:**
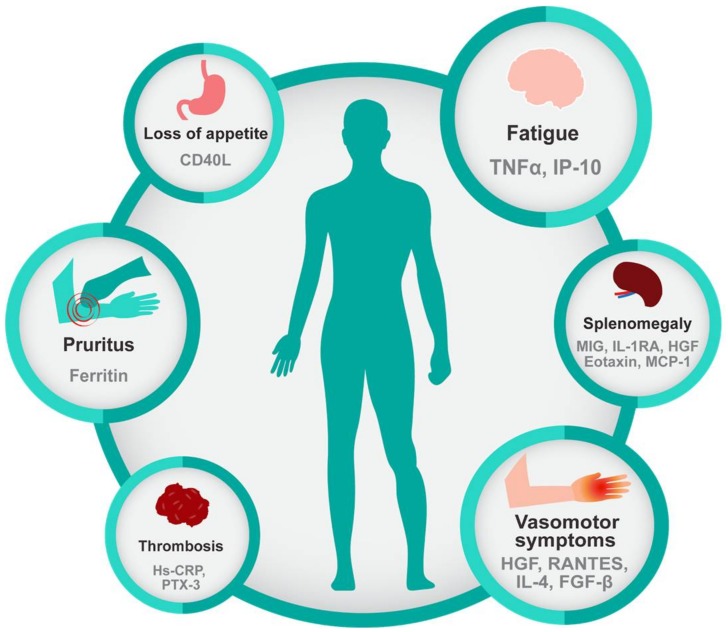
Inflammatory cytokines are correlated with characteristic features of myeloid malignancies. Common symptoms of myeloid malignancies, especially of primary myelofibrosis, include fatigue, pruritus, loss of appetite, and vasomotor symptoms and are significantly correlated with inflammatory biomarkers. Splenomegaly, which is inherent to the biology of MPN is also associated with increased inflammatory cytokines as is thrombosis a common complication of MPN. Referenced in [30,47,48].

MPN patients present a broad-spectrum of symptoms, even in early-stage disease, and vary among individuals, though some symptoms are more prevalent in ET, PV, or PMF [43]. Regardless of the subtype, the most common complaint is fatigue (80.7%), followed by pruritus (52.2%), night sweats (49.2%), bone pain (43.9%), and fever (13.7%) [30]. Interestingly, specific symptoms and complaints have been correlated with elevated levels of specific inflammatory biomarkers, as shown in Figure 2. Other symptoms such as abdominal discomfort, early satiety, and numbness/tingling in the extremities are also frequent complaints in MPN [30]. The recently developed National Comprehensive Cancer Center Network (NCCN) guidelines for PMF address the importance of symptom burden and recommend intervention to reduce symptom burden regardless of the prognosis scoring category. Symptom scoring tools such as the Myelofibrosis Symptom Assessment Form (MF-SAF) and the Myeloproliferative Neoplasm Symptoms Assessment Form (MPN-SAF) were developed in order to assess the clinical spectrum of physical symptoms that could affect the quality of life of MPN patients [44,45]. These tools were also used to objectively measure the impact of the JAK1/2-inhibitor, ruxolitinib, on symptom burden in the COMFORT-I trial [46]. Ruxolitinib, a potent anti-inflammatory drug, gained Food and Drug Administration (FDA) approval based on its ability to reduce spleen size and reduce symptom burden, which further highlights the central importance of symptoms in this disease. Treatment with Ruxolitinib resulted in reduction of multiple cytokines, including C-reactive protein (CRP), IL-1RA, macrophage inflammatory protein 1β (CCL4), TNFα, and IL-6 in PMF patients coincident with symptom improvement, further providing a link between inflammatory cytokines and symptom burden in MPN [9] (Figure 2).

### 2.4. Inflammatory Diseases and Cancers Share Therapies

The JAK/STAT signaling pathway plays a central role in hematopoiesis and the immune response, since constitutive activation of JAK/STAT signaling by gain-of-function mutations leads to the development of hematologic malignancies [49]. The discovery of the mutation JAK2^V617F^ in 2005 has revolutionized our understanding of MPN diseases and allowed the creation of new targeted therapies to the Janus kinase (JAK) [50,51,52,53,54]. JAK inhibitors are also useful in autoimmune disease such as alopecia areata [55], psoriasis [56] and graft versus host disease [57,58]. Tofacitinib, a JAK2/JAK3 inhibitor, is FDA-approved for methotrexate-resistant forms of rheumatoid arthritis and psoriatic arthritis. The efficacy of JAK inhibitors is currently being investigated on solid tumors such as pancreatic, lung, colon and breast cancers [59] as well as inflammatory bowel diseases [60,61]. 

One potential explanation for the observed “double duty” of drugs for hematologic malignancies and autoimmune disease is shared signaling pathways that activate immune signaling and also drive leukemogenesis. However, the fact that anti-inflammatory drugs work well in hematologic malignancies also implies that inflammation mediated by host normal immune cells could facilitate disease progression, and that the true utility of the drug is not by blocking activation of signaling in the neoplastic cell, but through blocking inflammatory signals in non-neoplastic immune cells. A prime example is the use of ruxolitinib in MPN. A recent clinical trial investigating a JAK1 inhibitor, itacitinib, in myelofibrosis demonstrated that this agent had similar efficacy to Ruxolitinib [62]. This shows that a drug devoid of JAK2 inhibition but that has the ability to reduce inflammatory cytokines through JAK1 inhibition has a similar utility as a JAK2 inhibitor in MPN. In addition, a small study with etanercept, a TNFα inhibitor, demonstrated an improvement in constitutional symptoms in myelofibrosis and showed only mild toxicity [63]. This suggests that much of the pathology in MPN may be driven by inflammation rather than the expansion of the neoplastic clone itself. Combining therapies that reduce inflammation and target the malignant clone is a rational approach towards a cure for MPN. Indeed, trials combining ruxolitinib and IFNα, which functions as an anti-inflammatory agent and specifically targets mutant cells, respectively, are underway and hold promise [64,65].

## 3. Inflammation Drives the Onset/Progression of Myeloid Malignancies

### 3.1. Inflammation Promotes Clonal Hematopoiesis

While substantial research has correlated chronic inflammation with myeloid malignancies, increasing evidence suggests that chronic inflammation may promote clonal hematopoiesis. Clonal hematopoiesis of indeterminate potential (CHIP), or simply clonal hematopoiesis (CH), is defined by the presence of cells in the blood or bone marrow with somatic mutations one or more of the 74 known mutations identified in hematological cancers. However, patients with CH have no overt phenotype of a hematological malignancy. Clonal hematopoiesis is an age-related condition and is correlated with increased incidence of non-hematological cancers [66] and atherosclerosis [67]. Clonal hematopoiesis can be found in over 10% of the population over the age of 70, and increases in prevalence with age [68]. Additionally, modern theories have attributed CHIP to being a significant cause of mortality and morbidity in developed countries, due to the pro-thrombotic nature of the disease course contributing to stroke and cardiovascular disease [67].

**Figure 3:**
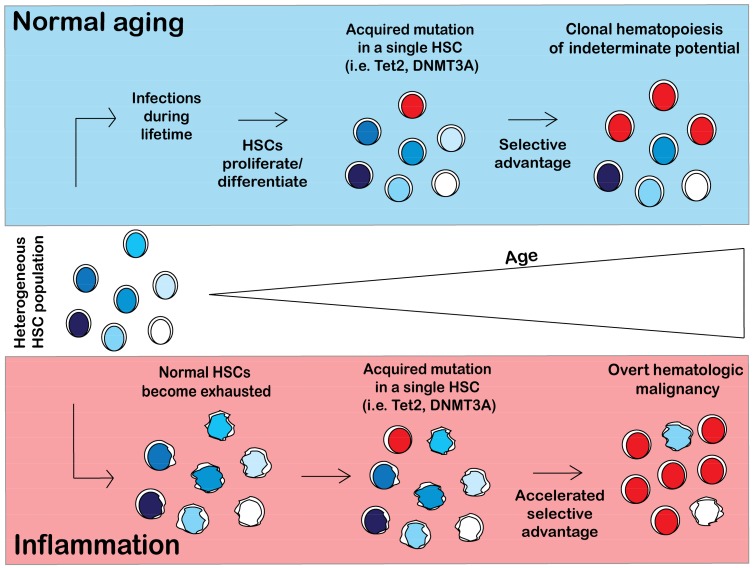
Model of clonal hematopoiesis in normal and inflammatory conditions. In a normal person, hematopoietic stem cells (HSCs) will differentiate and self-renew over the course of a lifetime to replenish the blood system. HSCs will acquire somatic mutations as a result of proliferative stress. Certain HSC mutant clones (i.e., Tet2, DNMT3A) will acquire a selective advantage over wild type (WT) HSCs, resulting in clonal hematopoiesis. However, in an inflammatory environment, normal HSCs are exhausted and have impaired fitness. HSCs will acquire somatic mutations and will likely have an accelerated selective advantage over the exhausted HSCs, resulting in an earlier onset of CHIP (not illustrated in figure) and possibly in hematologic malignancy.

Inflammation likely plays a contributory if not causal role in hastening the onset of CHIP, though the mechanism linking inflammation to CHIP still remains uncertain. The model of the pathogenesis of CHIP in normal aging conditions and in a chronic inflammatory environment is shown in Figure 3. Upon normal aging, a person will encounter multiple pathogens and will mount an immune response to fight infection, causing normal HSCs to differentiate and proliferate in order to replenish the body’s immune cells. Proliferative stress induces DNA damage and causes acquisition of somatic mutations in a single HSC. For CH to arise, a mutant HSC must have a selective advantage over the non-mutant stem cell pool. Given that CH is common in elders, development of clones with CH-related mutations may be an inevitable consequence of aging (Figure 3). 

### 3.2. Aging of Hematopoietic Stem Cells Is Mediated by Inflammation

During aging, human and mouse HSCs increase in frequency but have inferior self-renewal capabilities [69,70]. Aged HSCs also display myeloid skewed differentiation with a decline in lymphopoiesis [71,72]. Compared to younger HSCs, aged HSCs exhibit upregulation of genes associated with the stress response and inflammation and down-regulation of genes involved in the preservation of genomic integrity and chromatin remodeling [73]. In steady state, HSCs are mostly quiescent but in response to hematopoietic challenges such as blood loss, infection, or inflammation, HSCs exit from quiescence to proliferate and increase the output of mature hematopoietic cells [74]. HSC aging can be experimentally hastened by increasing the proliferative history of HSCs through serial transplantation [75,76] or stressing them with multiple injections of myeloablative chemotherapeutic regimens [77].

Chronic inflammation causes HSC aging and leads to HSC exhaustion. Chronic low dose exposure to lipopolysaccharide (LPS), a component of the membrane of gram negative bacteria, induces HSC to exit quiescence and cycle, increase reactive oxygen species (ROS), and accumulate DNA damage [78,79,80,81]. This proliferative stress leads to a reduction in HSC fitness and ultimately HSC exhaustion. Further, mice with sustained *Mycobacterium avium* infection exhibited severe HSC defects as a result of IFNγ-dependent terminal differentiation including pancytopenia and impaired self-renewal and engraftment [79]. HSC that are unable to sense inflammatory signals have superior competitive fitness over wild-type counterparts that retain the ability to respond to inflammatory stimuli [82]. Accelerated aging of HSC from chronic inflammation may promote the emergence of HSC with mutations that endow resistance to inflammation-induced aging (Figure 3). 

### 3.3. Selective Pressures Will Influence the Outgrowth of Specific Clones

**Figure 4:**
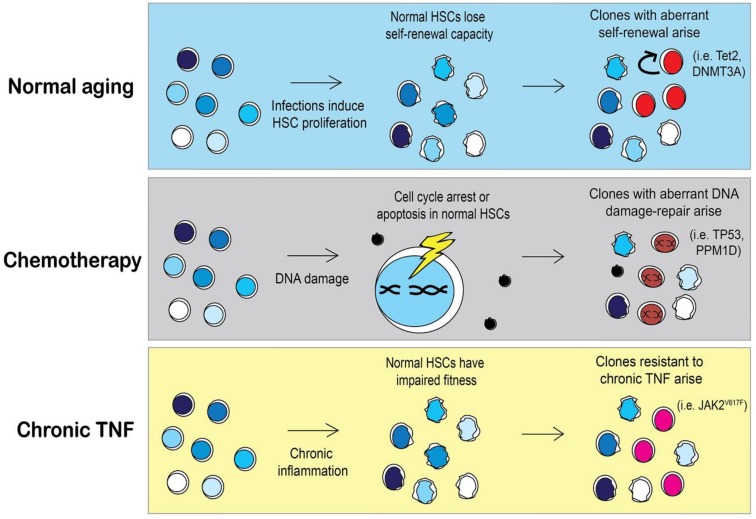
Selective pressures may shape clonal expansion of mutant HSCs. During aging HSCs lose their self-renewal capacity as the cells continuously replenish the blood system. As a result, HSCs with mutations that skew towards self-renewal, such as Tet2 or DNMT3A, will have a selective advantage over aged HSCs [84,86]. However, in a person that previously received chemotherapy, HSC clones with different mutations will arise. Chemotherapy induces DNA damage in HSCs and activates DNA damage repair pathways or apoptosis. The incidence of HSCs with mutations in DNA damage repair pathways such as TP53 or PPM1D is significantly higher in people with previous chemotherapy treatment [66]. Specific pressures may allow for the expansion of less common mutations such as JAK2^V617F^. MPN patients with JAK2^V617F^ mutations have elevated TNFα, contributing to the chronic inflammatory environment. Under chronic inflammation, normal HSCs will become exhausted and lose their competitive fitness, allowing for the emergence of mutant HSCs that are resistant to TNFα [85].

The same principles of natural selection imposed on species can also be applied to populations of cells [83]. Selective pressures from ecological niches fostering the emergence of adaptations in a species are analogous to pressures that occur in stressful hematopoietic microenvironment in which stem cells reside. Selective pressures can be encountered by genetic predispositions (autoimmune diseases), lifestyle (smoking), or therapies from earlier diseases (i.e., cytotoxic agents). The pressures upon an HSC pool dictate which clones will have a selective advantage and emerge, as illustrated in Figure 4. The most common mutations associated with CH are Tet2 and DNMT3A, which result in defects in HSC differentiation and increased self-renewal [84]. What remains to be seen is whether preserving the fitness of the normal HSC pool may prevent the emergence of neoplastic clones.

Given that clonal hematopoiesis can result from the outgrowth of one or more of 74 known driver genes of leukemia, there is still a paucity of information regarding the mechanism of the outgrowth of the more rare mutant clones, such as JAK2^V617F^. It is likely that less common HSC clones arise in a person in response to a very specific pressure. Similarly, the type of clonal mutation that a person with CH has is likely to inform us of the pressure that was put on their stem cell pool. For instance, JAK2 mutant HSCs are resistant to suppressive growth signaling from chronic TNFα [85]. However, chronic TNFα cannot be the only pressure to select for JAK2^V617F^ clones because TNFα is elevated in most myeloid malignancies (Figure 1). A complex environment of elevated cytokines may be required for the outgrowth of JAK2^V617F^, as represented in Figure 4. Cell extrinsic influences probably contribute to selective advantage of mutant clones, as it is unlikely that single clone produces enough inflammation to provide selective advantage over normal HSCs.

#### 3.3.1. Chemotherapy and Other DNA Damaging Agents as a Selective Pressure

It is well understood that previous exposure to DNA damaging agents such as chemotherapies or radiation increases the risk of a secondary hematologic malignancy [87]. However, there is growing interest into the exact mechanisms by which chemotherapy or radiotherapy induce secondary hematologic malignancies. One recent study showed that CH is present in approximately 25% of solid-tumor cancer patients and is associated with increased age, prior radiation, and smoking [66]. The incidence of CH was not higher in cancer patients who had previous exposure to chemotherapy over those who were not exposed to chemotherapy; this suggests that the mutant clones were already present at the time of cancer diagnosis and not from chemotherapy-induced mutations. However, in patients harboring CH mutations and who also had prior exposure to chemotherapy, there was a higher chance that these patients had TP53 or PPM1D mutations rather than DMNT3A or Tet2 clones [66]. This suggests that chemotherapy provides a selective advantage for certain types of HSC clones (i.e., TP53 or PPM1D) and not for other mutant clones (i.e., DNMT3A or Tet2). Another recent study investigated the influence of chemotherapy exposure on the variant allele frequency (VAF) of HSC clones in patients with a variety of CH mutations. In patients that received eight cycles of chemotherapy, the VAF of clones with a DNMT3A mutation remained unchanged after 20 months of treatment. However, the VAF of clones with mutations in the DNA damage-repair protein, RAD21, was increased following chemotherapy [88]. This study confirmed previous data in mouse models showing that DNMT3A mutant HSCs are resistant to anthracycline exposure stemming from an inability to sense and repair DNA torsional stress [89]. Collectively, these findings provide evidence that response of HSCs to DNA damaging agents is mutation dependent. It is possible that the types of mutations that a person with CH harbors may be informative of the selective pressures that were imposed on their HSCs over a person’s lifetime. 

Hydroxyurea, also known as hydroxycarbamide, is a common cytoreductive agent used in MPN. Hydroxyurea is converted to a free radical nitroxide (NO) in vivo and then transported by diffusion into cells where it inhibits DNA synthesis by inactivating ribonucleotide reductase. Although controversial, long-term use of hydroxyurea may potentially promote progression to leukemia [90]. It is possible that long-term exposure to hydroxyurea compounded with chronic inflammation selects for mutated HSC clones that result in leukemia. 

#### 3.3.2. Lifestyle Pressures That Influence Inflammation and Hematopoiesis

Lifestyle factors such as obesity and smoking are associated with myeloid malignancy development through the promotion of chronic inflammation. Elevated body mass creates an inflammatory environment and is a risk factor for myeloid malignancy development [91]. Diet-induced obesity modeled by a high fat diet (HFD) changes the composition of adipocyte tissue in the bone marrow and disrupts the ability of mesenchymal progenitors to generate osteoblastic cells [92,93,94]. Obesity directly harms HSCs, as shown in a HFD model as well as in db/db mice. HSCs from an obese mouse have an exacerbated proliferative response following transplantation, which leads to increased contribution in primary transplants, but negatively impacts competitive ability in secondary transplants [93]. These effects of obesity appear to be driven by upregulation of the gene *Gfi* that is driven by oxidative stress. The effects of obesity on HSC are not reversible with normalization of weight but can be prevented by the antioxidant N-Acetylcysteine [95]. Consistent with these findings is the increased risk of AML [96], MDS [39], and MPN [97] disease development among those with elevated body mass index (BMI) at baseline. 

Smoking is a second lifestyle factor that has been associated not only with elevated inflammation [98,99,100], but with disease development for AML, MDS, CMML and MPN [101,102,103,104]. Smoking induces oxidative stress and leads to a chronic inflammatory state, which may act synergistically to promote malignancy. In addition, smokers are more likely to have CHIP [66], suggesting that smoking may play an important role in the very early stages of myeloid malignancy development.

The microbiome is another parameter that affects inflammation and can be manipulated by lifestyle. The human microbiome is defined as the entirety of micro-organisms (including bacteria, viruses, fungi and parasites) living in the human body in a symbiotic interaction where they endorse a protective role most of the time. The microbiome performs digestive and metabolic functions and is responsible for 40% of the host’s energy intake [105]. Besides energy harvest, it plays a role in the development of the host’s immune system, as mice lacking gut microbes have altered immune responses [106]. Microbiota dysregulation (or dysbiosis) is seen in a variety of inflammatory diseases including inflammatory bowel disease [107], lupus [108] and rheumatoid arthritis [109]. Viruses and bacteria are also well-recognized triggers of hematologic conditions such as MALT lymphomas induced by *H. pylori* [110]. Thus, targeting or modulating the microbiota may be a novel therapeutic strategy that could complement established treatments for inflammatory conditions.

The microbiome directly modulates innate and adaptive immune responses. For example, germ-free mice infected with *Listeria monocytogenes* have reduced myelopoiesis and differentiation compared to colonized mice [111]. These infected mice die from systemic infection but recolonization of the gut microbiota from colonized mice afforded the mice resistance to infection [111]. Disruption of microbiota-host symbiosis has been implicated as a promoter of colorectal cancer through the induction of inflammatory cytokines by immune cells [112]. Pathobiotic bacteria invade normal colon tissue and induce local inflammation, creating an important link between the gut microbiota and colorectal cancer development [113]. Just as pathobiotic bacteria can induce inflammation and lead to colon cancer, beneficial symbiotic bacteria can reduce inflammation and protect the host from cancer.

Josefsdottir et al. demonstrated an instructive role for the microbiome in hematopoiesis. Depletion of the microbiome in mice with long-term broad-spectrum antibiotics led to impaired hematopoietic progenitor maintenance and granulocyte maturation that was a result of altered T cell homeostasis. The antibiotic induced defects were reversible with fecal microbiota transfer, demonstrating that preservation of the microbiome could be used to prevent antibiotic-associated bone marrow suppression [114]. New evidence of the interactions between the microbiome, inflammation and blood disorders are growing, opening a new promising field in cancer prevention and therapeutics.

### 3.4. The Bone Marrow Microenvironment and Inflammation

The role of the bone marrow microenvironment on HSC homeostasis has been of considerable interest for several decades. The BM niche involves a complex interaction of multiple cell types including HSCs and stromal cells such as mesenchymal (MSCs) and endothelial cells (ECs) to maintain steady-state hematopoiesis. In the context of inflammation, these interactions are even more critical as MSCs and ECs can detect inflammatory cytokines and engage an “inflammatory loop”, harming healthy HSCs and modifying regular hematopoiesis [115]. This inflammatory state was shown to trigger fibrosis and angiogenesis, contributing to the pathogenesis of MPN [116,117]. Other malignancies, such as myelodysplasia and secondary leukemias, can be induced by abnormal osteoprogenitors within the bone marrow niche [118]. Besides its possible role as a driver of the inflammation in hematologic malignancies, the bone marrow microenvironment could also potentiate relapse after chemotherapy, and may be a potential therapeutic target for treatment of resistant leukemias [119]. While it is well accepted that the bone marrow microenvironment plays a critical role in myeloid malignancies, it remains to be seen whether the microenvironment promotes the onset of clonal hematopoiesis.

## 4. Future Prospects in the Clinic and on the Bench 

There has been a rapid expansion of therapies for hematologic malignancies targeting signaling pathways co-opted by neoplastic cells that allow them to survive and expand. However, much less attention has been paid to the normal HSC pool and the selective pressures that allow for mutant clones to outcompete their wild-type “normal” counterparts. Interventions that preserve or even rejuvenate the fitness of the “normal” HSC pool may neutralize the selective pressure for the neoplastic clones. Chronic inflammation drives HSC aging and leads to a reduction in fitness. Therefore, interventions that reduce inflammation or protect HSC before mutant clones expand may be more impactful than eradicating mutant clones after they have expanded and taken over hematopoiesis. Low-risk interventions with lifestyle adjustments such as diet and exercise may be an ideal approach to reduce inflammation in those individuals with small mutant clones without clinical consequences (i.e., CHIP).

The factors that drive the initial emergence and progression of CHIP to a hematologic malignancy remain undefined, but inflammation likely plays a role in both of these processes. To date, most studies regarding CHIP have consisted of retrospective studies in humans. While mouse models of the most common CHIP associated mutations, including Tet2, DNMT3A, PPM1D, and JAK2^V617F^ exist, no models of spontaneously emerging CHIP are present. It is unclear whether CHIP is a common feature of murine hematopoietic aging as it is in humans. If so, it would be of interest to define the external or intrinsic factors that promote or prevent the emergence of CHIP. Mathematical modeling can be used as an approach to help define how specific pressures lead to the selective outgrowth of neoplastic HSC clones. Multiple investigators have modeled the effects of inflammation on MPN mutant versus wild-type HSCs, and this approach could be expanded to test the effect of other selective pressures on both types of HSCs [120,121]. 

## 5. Conclusions

Our understanding of the role of inflammation in myeloid malignancies continues to expand. In this review, we discussed development, progression and symptom burden of myeloid hematologic malignancies in the context of chronic inflammation. Chronic cytokine stimulation leads to HSC exhaustion and provides a selective advantage for tolerant mutant clonal stem cells. What remains to be seen, however, is how different types of selective pressures promote the outgrowth of mutated HSCs. Interventions that preserve HSC fitness through reduction of inflammation could potentially prevent initiation and/or progression of myeloid malignancies. Targeting inflammation could be as valuable as targeting the malignant clone, at least for chronic myeloid diseases.
